# A phase IV, multicentre, open‐label study of emicizumab prophylaxis in people with haemophilia A with or without FVIII inhibitors undergoing minor surgical procedures

**DOI:** 10.1111/hae.14574

**Published:** 2022-05-05

**Authors:** Miguel Escobar, Amy Dunn, Doris Quon, Ben Trzaskoma, Lucy Lee, Richard H. Ko, Shannon L. Carpenter

**Affiliations:** ^1^ University of Texas Health Science Center Houston Texas USA; ^2^ Nationwide Children's Hospital Columbus Ohio USA; ^3^ Orthopedic Hemophilia Treatment Center Los Angeles California USA; ^4^ Genentech, Inc. South San Francisco California USA; ^5^ Children's Mercy Hospital Kansas City Missouri USA

To the Editor:

People with haemophilia A (PwHA) often require general minor surgical procedures such as dental surgeries and endoscopies. In addition, central venous access device (CVAD) implantations or removals are common procedures associated with intravenous administration of clotting factors. Owing to the increased risk of prolonged bleeding, management of surgery is an important consideration for PwHA.^1^

Emicizumab is a bispecific humanized monoclonal antibody that bridges activated factor (F)IX and FX, substituting for the function of deficient activated FVIII in PwHA.^2^ Emicizumab prophylaxis has been shown to provide effective bleed control in PwHA with or without inhibitors in several phase III trials;^3^ however, these studies were not designed to specifically assess the use of emicizumab in PwHA undergoing surgery. Current guidelines on the management of PwHA do not include consistent guidance on PwHA undergoing surgery while receiving emicizumab prophylaxis.^1^

The present study was a phase IV, multicentre, single‐arm, open‐label study performed in PwHA of any age, with or without FVIII inhibitors (NCT03361137). PwHA were eligible to participate if they were receiving emicizumab (at minimum had completed the loading dose period [3 mg/kg per week for 4 weeks]), were scheduled to undergo a minor surgical procedure within 60 days of study enrolment and planned to receive emicizumab for ≥1 month following surgery (Supplementary Table S1).

The study was conducted in accordance with the ICH E6 guidance for Good Clinical Practice, the Declaration of Helsinki, and the ICH E2A guideline. Adult participants provided written informed consent prior to study‐related procedures; participants <18 years of age had informed consent provided by their legal guardian.

Surgical procedures from label‐enabling trials for FVIII products were reviewed. Procedures classified as minor in multiple studies, as well as any procedures deemed by the study Steering Committee to have the appropriate complexity and duration, were designated as minor procedures.

There was three primary efficacy‐related endpoints: (1) percentage of participants who had excessive bleeding at the surgical site and who required bypassing agents (BPAs)/FVIII for surgery‐related bleeding from the start of surgery until discharge from surgery; (2) percentage of participants who did not have excessive bleeding at the surgical site and who did not require BPAs/FVIII for surgery‐related bleeding from the start of surgery until discharge from surgery; (3) occurrence of bleeding and BPA/FVIII use after discharge from surgery. Excessive bleeding prior to discharge from surgery was defined according to the recommendations of the Scientific and Standardization Committee of the International Society on Thrombosis and Haemostasis and included ratings of fair‐to‐poor on the haemostatic response scale, which translates to blood loss of ≥25% over what would be expected for a person without haemophilia^4^; bleeding was assessed by the healthcare professional performing the surgery. Peri‐operative administration of BPAs, FVIII, and antifibrinolytics was at the discretion of the treating physician. Bleeding and BPA/FVIII use after discharge from surgery were self‐reported by the participants using a Bleeds and Medications Diary.

Safety endpoints included the incidence and severity of adverse events (AEs), serious AEs, and AEs of special interest; and the percentage of participants with complications requiring hospitalization or return to surgery. Exploratory endpoints included the percentage of participants with zero bleeds during the 28 days following surgery (inclusive of those at locations other than the surgical site), and emicizumab plasma concentrations on the day of surgery and relationship with outcome (plasma concentrations were not used to direct clinical decision making).

From 28 June 2018 to 13 March 2020, a total of 14 PwHA were enrolled (11 with FVIII inhibitors and three without) across eight sites in the US. Enrolment for this study was terminated early due to low numbers of patients undergoing minor surgeries and the limited variety of surgical procedures. Of the 14 participants, 13 underwent minor surgical procedures and were included in the analysis. Median (range) age was 11.0 (5–22) and 11.0 (3–36) years for participants with and without FVIII inhibitors, respectively (Supplementary Table S2).

Thirteen surgeries were performed during this study, comprising 11 CVAD removals and two simple dental extractions. Overall, 7/10 (70%) and 3/3 (100%) participants with and without FVIII inhibitors, respectively, did not have excessive bleeding at the surgical site and did not receive BPAs or FVIII post‐operatively. Only one participant experienced excessive bleeding during surgery, while four reported post‐operative bleeding. Three participants received treatment with recombinant activated FVII (rFVIIa) during surgery and three participants received rFVIIa post‐operatively (Figure 1).

**FIGURE 1:**
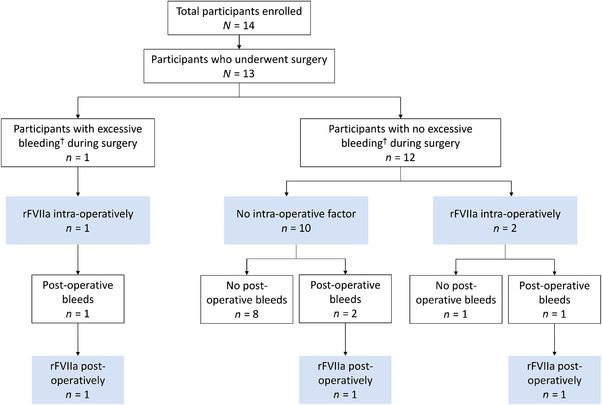
Summary of bleeding and use of coagulation factor during and after surgery. ^†^Excessive bleeding was defined as ratings of fair to poor on the haemostatic response scale^4^ and translates to blood loss of ≥25% over expectation for a participant without haemophilia, prior to discharge from surgery. rFVIIa, recombinant activated factor VII.

Two of the participants with FVIII inhibitors who underwent CVAD removal received rFVIIa during surgery, although they did not have excessive bleeding (Table 1). One of these participants was administered rFVIIa 15 min prior to surgery to prevent peri‐operative bleeding; this was documented as a protocol deviation as use of FVIII and BPAs in the 24 h prior to surgery was not permitted in this study. This participant also received tranexamic acid for 8 days following surgery. No intra‐ or post‐operative bleeding occurred. The other participant received a dose of rFVIIa immediately after the procedure, as more local swelling occurred than would be expected and there was concern about potential haematoma formation; however, a haematoma was not reported. After being discharged from surgery, this participant received one dose of rFVIIa for treatment of a post‐operative bleed.

**TABLE 1:** Surgery‐associated bleeding events and use of coagulation factor

**Participant**	**Procedure**	**Excessive bleeding during surgery** **^a^**	**rFVIIa or FVIII use during surgery** **^b^**	**Post‐operative bleeding** **^c^**	**rFVIIa or FVIII post‐operatively** ^b^, ^c^
**Participants with FVIII inhibitors**					
#1	Simple dental extraction	No	No	Yes	70.4 μg/kg rFVIIa
#2	CVAD removal	Yes	74.9 μg/kg rFVIIa	Yes	74.9 μg/kg rFVIIa
#3	CVAD removal	No	91.7 μg/kg rFVIIa	Yes	91.7 μg/kg rFVIIa
#4	CVAD removal	No	84.3 μg/kg rFVIIa	No	No
**Participants without FVIII inhibitors**					
#5	Simple dental extraction	No	No	Yes	No

Abbreviations: BPA, bypassing agent; CVAD, central access venous device; FVIII, factor VIII; rFVIIa, recombinant activated factor VII.

^a^ Excessive bleeding was defined as ratings of fair‐to‐poor on the haemostatic response scale^4^ and translates to blood loss of ≥25% over expectation for a participant without haemophilia, prior to discharge from surgery.

^b^ All doses of rFVIIa listed in the table correspond to single administrations.

^c^ Post‐operative bleeding and BPA/FVIII use were reported by the participant using the Bleed and Medication Diary.

A third participant with FVIII inhibitors, who underwent CVAD removal, experienced excessive bleeding during surgery and post‐operative bleeding at the surgical site (Table 1). This participant received one dose of rFVIIa intra‐operatively and one dose post‐operatively, and the bleeding resolved. Of the 11 participants who had CVADs removed, 7/9 (77.8%) participants with FVIII inhibitors and 2/2 (100.0%) participants without FVIII inhibitors had zero bleeds in the 28 days following discharge from surgery. Eight of these nine participants with zero bleeds received no BPAs or FVIII either during or after surgery (Figure 1).

Both participants who underwent dental extractions experienced post‐operative bleeding (Table 1). The participant without FVIII inhibitors did not receive post‐operative treatment with either FVIII or an antifibrinolytic. The participant with FVIII inhibitors received a single post‐operative dose of rFVIIa and two courses of oral treatment with aminocaproic acid.

Overall, three (23.1%) participants received antifibrinolytics peri‐operatively (Supplementary Table S3). No participant received activated prothrombin complex concentrate during the study.

Emicizumab plasma concentrations at the time of surgery and the days since last dose of emicizumab are presented in Supplementary Figure S1. The participant with FVIII inhibitors who experienced excessive bleeding during and after CVAD removal had an emicizumab plasma concentration of 15.9 μg/ml on the day of surgery. There were 2 days between the last dose of emicizumab and the surgery. This participant was adherent to a stable emicizumab maintenance dose of 40 mg weekly (approximately 1.5 mg/kg/week), with no missed doses reported and no breakthrough bleed occurring after the loading dose period, per the treating physician. The surgery was performed approximately 4 months after initiating emicizumab.

Most participants, 6/10 (60.0%) and 3/3 (100.0%) of those with and without FVIII inhibitors, respectively, did not report AEs (Supplementary Table S4). A total of six AEs were reported in four participants with FVIII inhibitors: headache (*n* = 2), constipation (*n* = 1), procedural pain (*n* = 1), adhesiolysis (*n* = 1), and haematoma (*n* = 1). The case of adhesiolysis was, in fact, a second surgical procedure to treat penile adhesions; however, this was listed as an AE in accordance with the study protocol. No serious AEs, thromboembolic events, thrombotic microangiopathies, or deaths were reported and no participant experienced complications that required hospitalization or return to surgery.

In this first and only prospective study to date of PwHA with or without FVIII inhibitors undergoing minor surgery while receiving emicizumab prophylaxis, most procedures were performed without the need for BPAs, FVIII treatment, or antifibrinolytics. One participant, who underwent CVAD removal, experienced bleeding intra‐ and post‐operatively, which was resolved with one dose of rFVIIa during surgery and a second dose post‐operatively. No other participants experienced excessive intra‐operative bleeding. The two participants who underwent dental extractions both experienced post‐operative bleeding, while most of those who underwent CVAD removal procedures had zero bleeds after discharge from surgery.

There was no apparent association between emicizumab concentration and efficacy; although the participant who experienced an intra‐operative bleed had a lower emicizumab concentration (15.9 μg/ml), conclusions cannot be drawn from a single case in a small study.

The findings of this study are consistent with previously published studies, including observations from the emicizumab clinical development programme, as well as real‐world data from patients undergoing minor surgery while receiving emicizumab prophylaxis.^5^, ^6^, ^7^, ^8^, ^9^, ^10^

No efficacy conclusions can be drawn from the present study due to study limitations that included: early enrolment termination due to a low number of PwHA undergoing minor surgical procedures, limited number of participants enrolled, and only two types of minor surgical procedures being evaluated.

In conclusion, common minor surgeries, such as CVAD removals and simple dental extractions, can be safely performed in PwHA with or without FVIII inhibitors receiving emicizumab prophylaxis. Clinical judgement should be exercised in determining management of PwHA undergoing minor surgical procedures, and a treatment plan coordinated by clinicians with expertise in the field and haemophilia treatment centres should be put in place prior to initiating surgery.

## DISCLOSURES

ME reports grants or contracts from Genentech, Inc.; honoraria for consulting from Magellan; honoraria for lectures from Biomarin, NovoNordisk, Takeda and Kedrion; participation on advisory boards for Genentech, Inc., Biomarin, NovoNordisk, CSL Behring, Sanofi, Takeda, Pfizer, Kedrion, UniQure and NHF. AD reports grants or contracts from Sanofi, Takeda, Freeline, Biomarin, ATHN and Novo Nordisk; consulting fees from Genentech, Inc., Kedrion, CSL Behring and Biomarin; payment or honoraria for lectures, presentations, speakers bureaus, manuscript writing or educational events from F. Hoffmann‐La Roche Ltd./Genentech, Inc.; participation on a Data Safety Monitoring Board or Advisory Board for UniQure and CSL Behring; leadership or fiduciary role in World Federation of Hemophilia USA. DQ reports payment or honoraria for lectures, presentations, speakers bureaus, manuscript writing or educational events from Biomarin, Bioverativ/Sanofi, NovoNordisk, Takeda and F. Hoffmann‐La Roche Ltd./Genentech, Inc.; participation on a Data Safety Monitoring Board or Advisory Board for Bayer, Biomarin, Bioverativ/Sanofi, Catalyst, NovoNordisk, Pfizer and F. Hoffmann‐La Roche Ltd./Genentech, Inc. BT, LL and RK are employees of Genentech, Inc. and hold stock or stock options as employees with Genentech, Inc./F. Hoffmann‐La Roche Ltd. SC reports payment or honoraria for lectures, presentations, speakers bureaus, manuscript writing or educational events from Genentech, Inc., Kedrion and NovoNordisk; leadership or fiduciary role in Hemostasis & Thrombosis Research Society and American Academy of Pediatrics.

## AUTHOR CONTRIBUTIONS

Amy Dunn, Doris Quon, Ben Trzaskoma, Richard H. Ko, and Shannon L. Carpenter contributed to the study design. Ben Trzaskoma and Lucy Lee contributed to the study conduct. Doris Quon and Ben Trzaskoma contributed to data collection. Miguel Escobar, Amy Dunn, Doris Quon, Ben Trzaskoma, Lucy Lee, Richard H. Ko, and Shannon L. Carpenter contributed to data analysis and interpretation. All authors revised the manuscript critically and provided final approval of the version to be published. All authors agree to be accountable for all aspects of the work.

## Supporting information

Supporting Information

## Data Availability

Data requests will be considered on a case‐by‐case basis. The data are not publicly available due to privacy or ethical restrictions.
