# Supplementary material for: A phase IV, multicentre, open‐label study of emicizumab prophylaxis in people with haemophilia A with or without FVIII inhibitors undergoing minor surgical procedures
Source: Haemophilia. 2022 May 5;28(4):e105–8. doi: 10.1111/hae.14574 (PMC9544354; doi:10.1111/hae.14574)
Supplement: Supplementary file 1 — Supporting Information [file HAE-28-e105-s001.docx]

**SUPPLEMENTARY MATERIAL**

**Supplementary Table S1.** Study inclusion and exclusion criteria

| **Inclusion** | **Exclusion** |
| --- | --- |
| - Written informed consent/assent form - Any age (newborn and older) - Ability to comply with the study protocol, according to the investigator’s judgment - For PwHA with inhibitors: diagnosis of haemophilia A and current or history of FVIII inhibitors (≥0.6 BU) and currently using BPAs for breakthrough bleeds - For PwHA without inhibitors: diagnosis of haemophilia A and no history of FVIII inhibitors (<0.6 BU), or history of FVIII inhibitors that have been tolerized for >5 years and using FVIII for breakthrough bleeds - Plan to receive at least the loading doses of emicizumab (3 mg/kg weekly for four weeks) and be adherent to emicizumab prophylaxis by the time of the surgery - Undergoing minor surgery within 60 days of study enrolment.^†^ Examples include:   - CVAD procedures (e.g. removal)   - Simple dental extractions   - Colonoscopy, cystoscopy, or endoscopy with biopsy   - Excisional skin biopsy - Plan to continue emicizumab prophylaxis 1.5 mg/kg weekly, 3 mg/kg every two weeks, or 6 mg/kg every four weeks for ≥1 month after surgery - For women of childbearing potential, agreement to remain abstinent or use contraceptive methods that result in a failure rate of <1% per year during the study period | - Diagnosis of a bleeding disorder other than haemophilia A - Tolerized to FVIII products (for PwHA with FVIII inhibitors) - Using FVIII products to treat breakthrough bleeds (for PwHA with inhibitors) - Tolerized to FVIII products for <5 years (for PwHA without inhibitors) - Treatment with BPAs or FVIII within 24 hours prior to surgical procedure - Undergoing a major surgical procedure - Previous (≤12 months prior) or current treatment for thromboembolic disease (with the exception of previous catheter-associated thrombosis for which anti-thrombotic treatment is not currently ongoing) or current signs of thromboembolic disease - Other conditions (e.g., certain autoimmune diseases, including, but not limited to diseases such as systemic lupus erythematosus, inflammatory bowel disease, and antiphospholipid syndrome) that may increase the risk of bleeding or thrombosis - PwHA who are at high risk for TMAs (e.g., have a previous medical or family history of TMAs), according to the investigator’s judgment - Known HIV infection with CD4 count <200 cells/μL within 24 weeks prior to enrolment - Pregnant or lactating, or intending to become pregnant during the study - PwHA who would refuse treatment with blood or blood products, if necessary - Any serious medical condition or abnormality in clinical laboratory tests that, in the investigator’s judgment, precludes the patient’s safe participation in and completion of the study - History of clinically significant hypersensitivity associated with monoclonal antibody therapies or components of the emicizumab injection - Treatment with any of the following:   - An investigational drug to treat or reduce the risk of haemophilic bleeds within 5 half-lives of last drug administration before Day 1   - A non-haemophilia related investigational drug within the last 30 days or 5 half-lives before Day 1, whichever is longer   - An investigational drug concurrently |

^†^Procedures classified as minor in multiple label-enabling studies, as well as any procedures deemed by the study Steering Committee to have the appropriate complexity and duration, were designated as minor procedures. Other minor surgical procedures could be included upon consultation and approval of the medical monitor.

BPAs, bypassing agents; BU, Bethesda units; CVAD, central venous access device; FVIII, factor VIII; HIV, human immunodeficiency virus; PwHA, people with haemophilia A; TMAs, thrombotic microangiopathies.

**Supplementary Table S2.** Baseline characteristics and demographics

| **All-enrolled population** | **Participants with FVIII inhibitors (N = 11)** | **Participants without FVIII inhibitors (N = 3)** |
| --- | --- | --- |
| **Age, years**  Median (range)  <18, n (%) ≥18–<65, n (%) | 11.0 (5–22)  9 (81.8) 2 (18.2) | 11.0 (3–36)  2 (66.7) 1 (33.3) |
| **BMI, kg/m^2^ (mean [SD])** | 20.5 (6.4) | 20.3 (3.7) |
| **Ethnicity, n (%)**  Hispanic or Latino Not Hispanic or Latino | 4 (36.4) 7 (63.6) | 1 (33.3) 2 (66.7) |
| **Race, n (%)**  Asian Black or African American White Multiple Unknown | 1 (9.1) 1 (9.1) 6 (54.5) 2 (18.2) 1 (9.1) | 0 1 (33.3) 1 (33.3) 0 1 (33.3) |
| **Surgical procedure, n (%)**  Removal of CVAD Simple dental extraction Not performed within study dates due to COVID-19 | 9 (81.8) 1 (9.1)  1 (9.1)^†^ | 2 (66.7) 1 (33.3) 0 |

^†^This participant did not undergo surgery within the scheduled dates and was therefore not included in the efficacy or safety analyses.
BMI, body mass index; CVAD, central venous access device; FVIII, factor VIII; SD, standard deviation.

**Supplementary Table S3.** Peri-operative antifibrinolytic treatment

| **Antifibrinolytic** | **Dose** | **Duration** | **Surgical procedure** | **Bleeding during surgery or post-operative bleeding**^†^ |
| --- | --- | --- | --- | --- |
| **Participants with FVIII inhibitors** |  |  |  |  |
| Aminocaproic acid | 53 mg/kg QID  80 mg/kg QID | 4 days  7 days | Simple dental extraction | Post-operative bleed requiring rFVIIa |
| Tranexamic acid | 1300 mg TID | 8 days | CVAD removal | N/A |
| **Participants without FVIII inhibitors** |  |  |  |  |
| Aminocaproic acid | 28 mg/kg QID | 8 days | CVAD removal | N/A |

^†^Bleeding during surgery was defined as ratings of fair-to-poor on the haemostatic response scale (Blanchette et al. J Thromb Haemost. 2014) and translates to blood loss of ≥25% over expectation for a participant without haemophilia, prior to discharge from surgery; post-operative bleeding was reported by the participant using the Bleed and Medication Diary.

CVAD, central venous access device; FVIII, factor VIII; N/A, not applicable; QID, four times a day; rFVIIa, recombinant activated factor VII; TID, three times a day.

**Supplementary Table S4.** Summary of safety in participants with or without FVIII inhibitors

|  | **Participants with FVIII inhibitors (N = 10)** | **Participants without FVIII inhibitors (N = 3)** |
| --- | --- | --- |
| **Any AE**^†^ | 4^‡,§^ | 0 |
| **Treatment-related AE** | 0 | 0 |
| **Grade 3–5 AE** | 0 | 0 |
| **AE leading to dose modification or interruption** | 0 | 0 |
| **AE leading to withdrawal from treatment** | 0 | 0 |
| **Serious AE**  Thromboembolic event  Thrombotic microangiopathy | 0  0  0 | 0  0  0 |
| **Complication requiring hospitalization or return to surgery** | 0 | 0 |

^†^AEs were documented until study completion at 28 days after surgery.

^‡^Events comprised headache (n = 2), constipation (n = 1), procedural pain (n = 1), adhesiolysis (n = 1), haematoma (n = 1). The adhesiolysis was a secondary procedure that was performed simultaneously with a CVAD removal; this was captured as an AE rather than a secondary procedure, as per the study protocol. The haematoma that occurred in one participant was present in the thenar eminence muscles of the hand and was classified as an AE; this was distinguished as an AE, in contrast to all other bleeding events, as it did not occur at the site of the CVAD removal.

^§^One participant with FVIII inhibitors did not undergo surgery during the study period and was excluded from the analyses; this participant experienced 4 AEs: headache, limb injury, pyrexia and device occlusion; none of the AEs were classed as treatment-related, grade 3–5 or serious and none resulted in dose modification or interruption or withdrawal from treatment.

AE, adverse event; CVAD, central venous access device; FVIII, factor VIII.

**Supplementary Figure S1.** Emicizumab plasma concentrations at time of surgery and days since last dose of emicizumab in participants with or without FVIII inhibitors


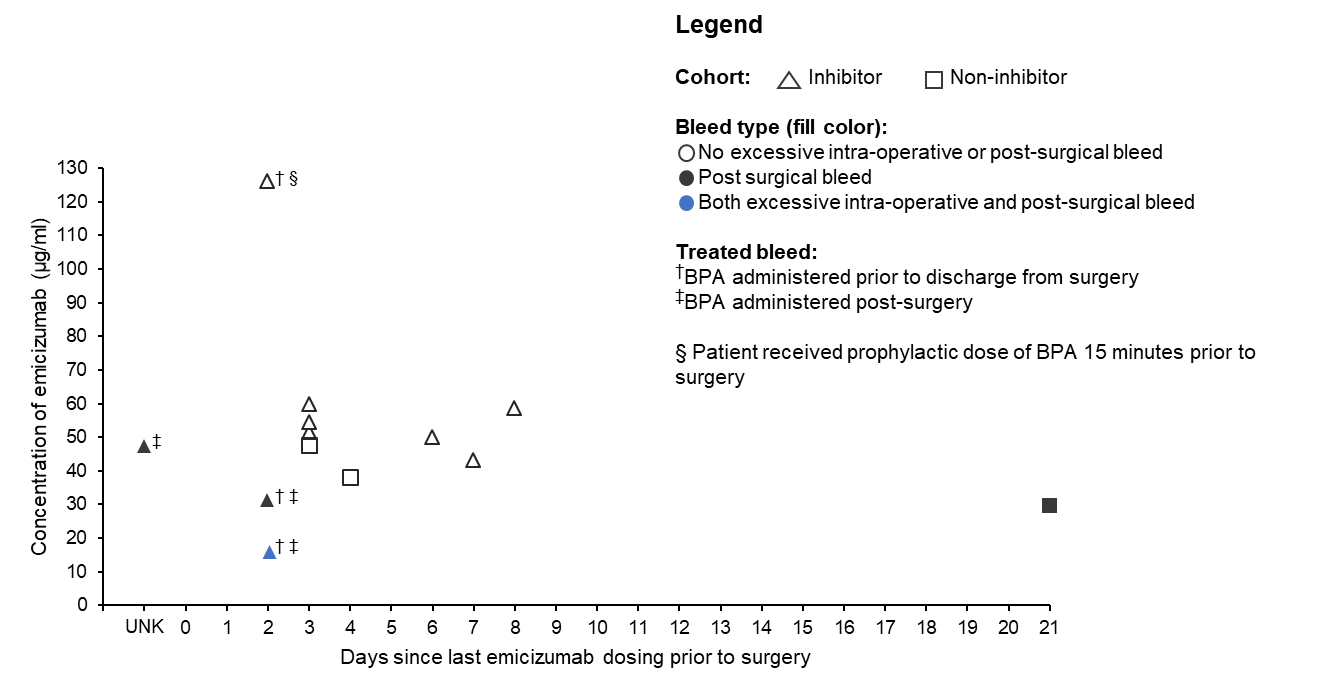


BPA, bypassing agent; FVIII, factor VIII; UNK, unknown.
